# Risk of All-Cause Mortality in Levothyroxine-Treated Hypothyroid Patients: A Nationwide Korean Cohort Study

**DOI:** 10.3389/fendo.2021.680647

**Published:** 2021-05-13

**Authors:** Seo Young Sohn, Gi Hyeon Seo, Jae Hoon Chung

**Affiliations:** ^1^ Division of Endocrinology and Metabolism, Department of Internal Medicine, Myongji Hospital, Hanyang University College of Medicine, Goyang, South Korea; ^2^ Department of Healthcare Review and Assessment Committee, Health Insurance Review and Assessment Service, Wonju, South Korea; ^3^ Division of Endocrinology and Metabolism, Department of Medicine and Thyroid Center, Samsung Medical Center, Sungkyunkwan University School of Medicine, Seoul, South Korea

**Keywords:** hypothyroidism, mortality, levothyroxine, cohort study, cardiovascular disease

## Abstract

**Background:**

Although hypothyroidism is associated with various comorbidities, its relationship with increased all-cause mortality remains controversial. The aim of this nationwide retrospective cohort study was to investigate whether hypothyroid patients treated with levothyroxine had increased mortality compared to controls.

**Methods:**

Hypothyroid subjects were identified through the Korean National Health Insurance Service Claims database between 2008 and 2017. Hypothyroidism in this study was defined as overt hypothyroidism treated with long-term prescription of levothyroxine (>6 months). After 1:3 age-, sex- and index year-matching, 501,882 patients with newly diagnosed hypothyroidism and 1,505,646 controls without hypothyroidism were included.

**Results:**

During a mean follow-up of 6 years, 25,954 (5.2%) hypothyroid patients and 59,105 (3.9%) controls died. Hypothyroidism was significantly associated with increased all-cause mortality (adjusted hazard ratio [HR], 1.14; 95% confidence interval [CI] 1.12–1.16) even with levothyroxine treatment. When stratified by age, sex, and cardiovascular disease risk, independent associations between hypothyroidism and mortality remained significant in all subgroups. The risk of mortality was higher in the < 65 age group (HR: 1.25, 95% CI: 1.22–1.29), men (HR: 1.28, 95% CI: 1.25–1.31), and the high cardiovascular disease risk group (HR: 1.31, 95% CI: 1.29–1.34). The mortality rate of hypothyroid patients was highest within 1 year of treatment and decreased with time.

**Conclusion:**

This nationwide, population-based cohort study showed that all-cause mortality was significantly higher in levothyroxine-treated hypothyroid patients than in non-hypothyroid controls. This association remained significant regardless of age, sex, and cardiovascular disease risk.

## Introduction

Hypothyroidism is a common endocrine disease, and its incidence and prevalence vary depending on the population being studied (1–3). In Korea, the reported incidence and prevalence of hypothyroidism, when defined as overt hypothyroidism with thyroid hormone prescription of > 60 days, were 2.26 and 14.28 per 1,000 individuals, respectively (2).

The etiology of hypothyroidism includes various conditions. Chronic autoimmune thyroiditis is the major subtype of hypothyroidism in iodine-sufficient areas of the world (4). However, non-autoimmune hypothyroidism has been reported as the most common cause of hypothyroidism in Korea (5). Regardless of cause, hypothyroidism is associated with a number of well-characterized metabolic changes, such as hyperlipidemia, hypertension, and coagulopathy, as well as endothelial dysfunction and cardiovascular disorders (6–10), all of which could theoretically lead to increased mortality. However, studies of hypothyroidism have yielded considerable variation in mortality data (11–24). Some studies have found an increased risk of mortality in hypothyroid patients (12–14, 16, 17, 19, 21–24), but others have not (11, 15, 18, 20). Possible reasons for such inconsistencies may be heterogeneity between studies in terms of definition and severity of hypothyroidism, characteristics of participants, selection of the control population, and controls for comorbidities.

In most patients, hypothyroidism is permanent and requires lifelong treatment. Standard treatment involves the administration of thyroid hormones to maintain serum thyroid-stimulating hormone (TSH) levels within the reference range (25). Most patients become clinically euthyroid once TSH levels return to normal. However, recovery of TSH levels might not reflect the normalization of hypothyroidism-mediated metabolic changes (26, 27), suggesting that mortality risk may persist in hypothyroid patients even with treatment.

To date, a number of cohort studies and meta-analyses have investigated the relationship between hypothyroidism and mortality (11–24, 28–38). However, most have been restricted to subjects with subclinical hypothyroidism (17, 20, 21, 28, 29, 33, 34, 38) and some studies have defined hypothyroidism based on only one TSH level measurement (13, 15, 22, 28, 30, 37). Therefore, these studies may not represent patients with hypothyroidism who require long-term thyroid hormone replacement.

In this nationwide retrospective cohort study, we investigated all-cause mortality risks of hypothyroid patients undergoing long-term levothyroxine therapy compared to age- and sex-matched control subjects.

## Methods

### Data Source

This nationwide retrospective cohort study was based on the Korean National Health Insurance (NHI) database provided by the Health Insurance Review and Assessment Service (HIRA). The NHI is the only public medical insurance system operated by the Korean government and membership is compulsory. To claim payments for patient care, all clinics and hospitals in Korea are required to submit data including personal identification number, diagnosis, and prescription information to the HIRA. Therefore, the HIRA database includes claims for the entire South Korean population and comprises patient demographics, diagnosis information based on the International Classification of Diseases, 10^th^ Revision (ICD-10) codes, all inpatient and outpatient claims data, interventions, and prescriptions. HIRA de-identifies patient data in accordance with the Act on the Protection of Personal Information Maintained by Public Agencies.

### Study Population

**Figure 1:**
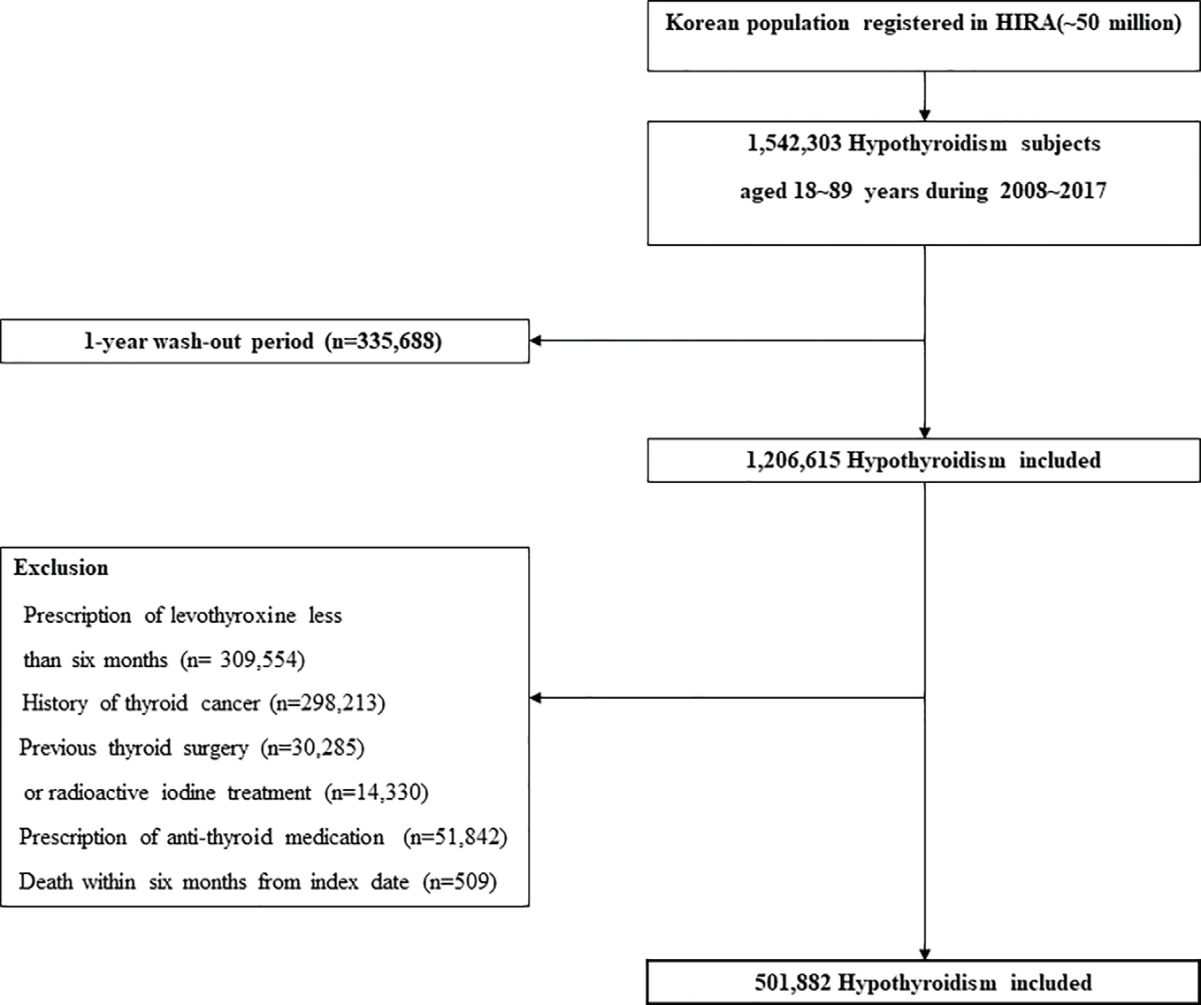
Selection of the hypothyroidism cohort.

**Hypothyroidism cohort.** In this study, claims data for levothyroxine prescriptions from 2008 to 2017 were evaluated to define the incidence of hypothyroidism. Hypothyroidism in this study was defined as overt hypothyroidism with long-term prescription of levothyroxine (>6 months). Only subjects aged 18–90 years at the time of prescription were included. A one-year washout period was applied to newly diagnosed cases of hypothyroidism. When a thyroid hormone is prescribed, it is usually repeated within one year in clinical practice. Therefore, we only included patients with a minimum washout period of 1 year. The washout period was defined as the period between January 2007 and the date of first prescription (the “index date”). The exclusion criteria were as follows: (i) a diagnosis of thyroid cancer at any time or a history of thyroidectomy or radioactive iodine treatment to exclude any effects of iatrogenic thyrotoxicosis; (ii) short-term prescription of thyroid hormones (<180 days) to avoid inclusion of transient hypothyroidism; and (iii) death within 6 months from the index date. Selection of the hypothyroidism cohort is illustrated in **Figure 1**.

**Control cohort.** Data for non-hypothyroid subjects (the control cohort) were also retrieved from the HIRA database. Individuals who received thyroid hormones or had a diagnosis of thyroid dysfunction-related diseases (ICD-10 code: E03.8, E03.9, E06.3) during the study period were excluded. In addition, the same exclusion criteria as in the hypothyroidism cohort were applied before matching the hypothyroidism cohort. Each hypothyroidism case was then matched to three non-hypothyroidism control individuals (1:3 matching) based on age, sex, and index date.

### Comorbidity

Comorbidities were mainly selected based on metabolic abnormalities related to hypothyroidism. Hypothyroidism is closely associated with cardiovascular risk factors that lead to cardiovascular events (6–10). Decreased hemodynamics in the circulatory system also lead to declines in glomerular filtration rate (GFR) in severe hypothyroidism (39). As cancer is the most common cause of death in Korea, we adjusted for the presence of malignancy because the primary endpoint was all-cause mortality (40).

Baseline comorbidities were identified according to their respective ICD-10 codes: diabetes mellitus (E10-E14), hypertension (I10), congestive heart failure (I50), myocardial infarction (MI) (I21, I22, I25.2), stroke (I60-I64, I69), chronic kidney disease (N17-N19), and malignancy (C code). A pre-existing comorbidity was defined as a disease diagnosed in at least three outpatient visits in the 1-year period preceding the index date. In cases of MI, stroke, and heart failure, inpatient hospitalization with records of any of their corresponding ICD codes as primary diagnosis was also defined as comorbidities.

### Mortality

The primary outcome of this study was all-cause mortality. Mortality was recorded in the NHI cohort based on the database of the Ministry of Public Administration and Security, which compulsorily receives all reports on deaths through official death notices. Cases and times of death from inception to December 31, 2018, were identified for all subjects.

### Statistical Analysis

The chi-square test was used to compare proportions and categorical variables between the hypothyroid and control cohorts. Student’s *t* test was used for comparisons of mean age between two groups. Crude incidence rates for mortality were calculated as the number of deaths per 1000 person-years. The incidence rates were further stratified by age (<65 and ≥65 years), sex, and cardiovascular disease (CVD) risk (high CVD vs. low CVD risk). High CVD risk was defined as the presence of hypertension, diabetes mellitus, or prevalent CVD (MI, stroke, and heart failure). Kaplan–Meier curves were used to describe and compare cumulative survival rates between the hypothyroid and control cohorts. The follow-up period started on the index date (date of first prescription of thyroid hormone) and was censored on the date of death or at the end of the study (December 31, 2018).

The Cox proportional hazard model was used to explore independent associations between hypothyroidism and mortality risk, and hazard ratios (HRs) were computed with 95% confidence intervals (CI). We conducted multivariable adjustments for age, sex, and comorbidities that affect mortality. Sensitivity analyses were performed in three groups according to the follow-up duration (<1 year, ≤1 to <3 years, and >3 years) from initial treatment with levothyroxine. All statistical analyses were conducted using R (version 4.0.2).

## Results

### Baseline Characteristics of the Cohort

A total of 501,882 patients with hypothyroidism and 1,505,646 controls treated between January 2008 and December 2017 were included. Baseline characteristics of the subjects are presented in **Table 1**. The mean age was 50.6 years, and women were predominant (82.6%). Hypothyroid patients had a significantly higher prevalence of all selected comorbidities than the control cohort. The mean follow-up durations were 71.6 [standard deviation (SD), 35.6] months and 72.1 (SD, 35.4) months for the hypothyroid and control cohorts, respectively.

**Table 1:** Baseline characteristics of the study populations.

	Hypothyroidism cohort	Control cohort	*P*-value
Total	501,882	1,505,646	
Male	87,233 (17.4%)	261,699 (17.4%)	0.99
Female	414,649 (82.6%)	1,243,947 (82.6%)	
Age			
mean ± SD	50.6 ± 15.0	50.6 ± 15.0	
<65 years	404,230 (80.5%)	1,212,690 (80.5%)	0.99
≥65 years	97,652 (19.5%)	292,956 (19.5%)	
Days of prescription			
<2 years	191,185 (38%)		
≤2 to <4 years	111,937 (22%)		
≤4 to <6 years	78,332 (16%)		
≤6 years	120,428 (24%)		
Comorbidities			
Hypertension	119,420 (23.8%)	299,126 (19.9%)	<0.001
Diabetes	62,441 (12.4%)	123,070 (8.2%)	<0.001
Myocardial infarction	3,303 (0.7%)	5,818 (0.4%)	<0.001
Stroke	18,186 (3.6%)	42,165 (2.8%)	<0.001
Heart failure	8,901 (1.8%)	12,737 (0.8%)	<0.001
Renal failure	8,370 (1.7%)	5,998 (0.4%)	<0.001
Malignancy	20,997 (4.2%)	33,485 (2.2%)	<0.001

SD, standard deviation.

### Impact of Hypothyroidism on the All-Cause Mortality

**Figure 2:**
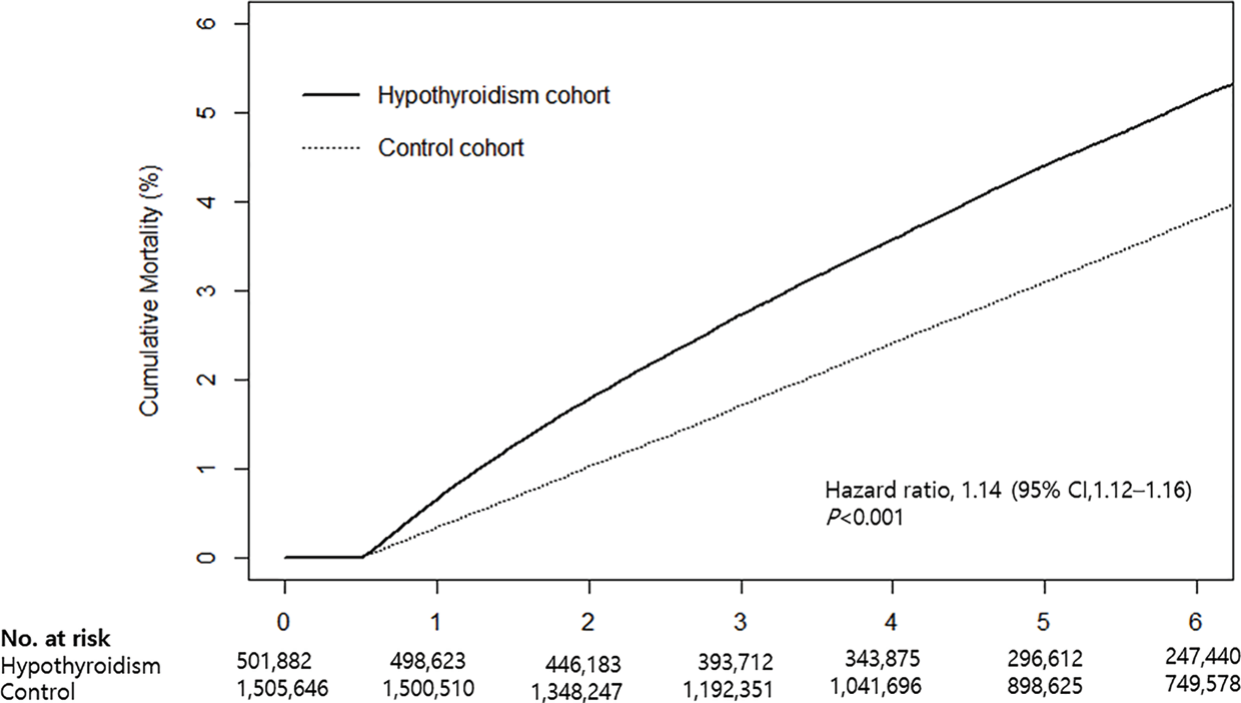
Kaplan-Meier curves for cumulative mortality in hypothyroid patients and the control cohort.

Cumulative mortality risk is illustrated in **Figure 2**. The mortality rate of hypothyroid patients was higher than that of the control cohort (**Table 2**). During the follow-up period, 25,954 (5.2%) hypothyroid patients died, compared with 59,105 (3.9%) controls. The crude mortality rate among hypothyroid patients was 8.66 per 1,000 person-years, compared with 6.53 per 1,000 person-years in the control cohort. When stratified by age, sex, and CVD, the crude mortality rate was consistently higher in all subgroups of the hypothyroid cohort.

**Table 2:** Incidence rates and hazard ratios for mortality in the hypothyroidism cohort and the control cohort.

	Hypothyroidism cohort		Control cohort		Hazard ratio for mortality	
Subgroup	Number of deaths	Mortality rate [CI] (per 1,000 person-years)	Number of deaths	Mortality rate [CI] (per 1,000 person-years)	Crude HR* [CI]	Adjusted HR** [CI]
All	25,954	8.66 [8.33–9.00]	59,105	6.53 [6.37–6.70]	1.33 [1.31–1.35]	1.14 [1.12–1.16]
Male	11,470	24.44 [23.04–25.90]	23,211	16.03 [15.38–16.69]	1.53 [1.49–1.56]	1.28 [1.25–1.31]
Female	14,484	5.73 [5.44–6.04]	35,894	4.72 [4.57–4.88]	1.21 [1.19–1.24]	1.06 [1.04–1.09]
Age groups						
<65 years	8,649	3.49 [3.26–3.73]	16,275	2.18 [2.07–2.29]	1.60 [1.56–1.64]	1.25 [1.22–1.29]
≥65 years	17,305	33.69 [32.12–35.32]	42,830	27.25 [26.44–28.07]	1.24 [1.22–1.26]	1.08 [1.06–1.10]
Comorbidities						
Low CVD risk group	8,111	3.64 [3.40–3.90]	25,157	3.52 [3.39–3.66]	1.03 [1.01–1.06]	1.10 [1.08–1.13]
High CVD*** risk group	17,843	23.19 [22.13–24.30]	33,948	17.81 [17.22–18.42]	1.30 [1.28–1.33]	1.31 [1.29–1.34]

CI, confidence interval; CVD, cardiovascular disease.

*Reference group is the control cohort.

**Adjusted for age, sex, and comorbidities in subgroup analysis. Sex subgroup is adjusted for age and comorbidities. Age subgroup is adjusted for sex and comorbidities. CVD risk subgroup is adjusted for age and sex.

***High CVD risk group is defined as the presence of hypertension, diabetes mellitus, or prevalent CVD (MI, stroke, and heart failure).

Cox proportional hazards analyses revealed that the mortality risk in hypothyroid subjects was higher than that in controls. The crude HR was 1.33 (95% CI: 1.31–1.35) and adjusted HR was 1.14 (95% CI: 1.12–1.16) (**Table 2**). When stratified by age, sex, and CVD risk, an independent association between hypothyroidism and mortality remained significant in all subgroups. The risk of mortality was higher in the <65 age group (HR: 1.25, 95% CI: 1.22–1.29), men (HR: 1.28, 95% CI: 1.25–1.31), and the high CVD risk group (HR: 1.31, 95% CI: 1.29–1.34) than in subgroups ≥65 years (HR: 1.08, 95% CI: 1.06–1.10), women (HR: 1.06, 95% CI: 1.04–1.09), and low CVD risk (HR: 1.10, 95% CI: 1.08–1.13).

In sensitivity analyses according to follow-up duration, mortality risk increased in the hypothyroid cohort at <1 year, ≤1–3 years, and ≥3 years after initial treatment with levothyroxine, but this association tended to decrease as the follow-up time increased (**Table 3**). The mortality rate in hypothyroid patients was the highest within 1 year of treatment and decreased with time.

**Table 3:** Hazard ratios for all-cause mortality stratified by follow-up duration after initial treatment.

	Adjusted HR* [CI]			
Subgroup	Total	<1 year	≤1–<3 year	≥3 years
All	1.14 [1.12–1.16]	1.50 [1.44–1.57]	1.27 [1.24–1.30]	1.01 [0.99–1.03]
Male	1.28 [1.25–1.31]	1.82 [1.71–1.95]	1.43 [1.38–1.49]	1.08 [1.04–1.11]
Female	1.06 [1.04–1.09]	1.28 [1.20–1.37]	1.17 [1.13–1.21]	0.98 [0.96–1.01]
Age				
<65 years	1.25 [1.22–1.29]	1.81 [1.66–1.97]	1.53 [1.46–1.60]	1.05 [1.01–1.09]
≥65 years	1.08 [1.06–1.10]	1.37 [1.30–1.44]	1.16 [1.12–1.19]	0.98 [0.96–1.01]
Comorbidities				
Low CVD risk group	1.10 [1.08–1.13]	1.57 [1.44–1.70]	1.30 [1.24–1.36]	0.96 [0.93–0.99]
High CVD** risk group	1.31 [1.29–1.34]	1.72 [1.63–1.81]	1.45 [1.40–1.49]	1.15 [1.12–1.18]

CI, confidence interval; CVD, cardiovascular disease.

*Adjusted for age, sex, and comorbidities in subgroup analysis. Sex subgroup is adjusted for age and comorbidities. Age subgroup is adjusted for sex and comorbidities. CVD risk subgroup is adjusted for age and sex.

**High CVD risk group is defined as the presence of hypertension, diabetes mellitus, or prevalent CVD (MI, stroke, and heart failure).

## Discussion

In this nationwide, retrospective population-based cohort study, we found that hypothyroidism was associated with an increased risk of all-cause mortality. This association remained significant regardless of age, sex, and CVD risk.

Many previous studies have investigated the link between hypothyroidism and mortality risk, but their results have been inconsistent (11–24, 28–34). Possible reasons for such discrepancies include considerable variations in the definition and severity of hypothyroidism, characteristics of study populations, and adjustments for comorbidities. Many studies have defined hypothyroidism based on a single measurement of TSH level and have used different reference ranges (13, 15, 22, 28, 30, 37), which carries the possibility of phenotype misclassification, as spontaneous normalization of an abnormally elevated TSH may occur in a substantial proportion of individuals (41). With regard to study populations, studies based on hospitalized patients have indicated a positive correlation between serum TSH concentration and mortality risk (13, 42), whereas no increased mortality risk has been reported in hypothyroid subjects in primary care settings compared with the euthyroid population (11, 15, 20). In addition, some studies did not adequately adjust for comorbid conditions (11, 15, 24, 42). Our definition of hypothyroidism, as individuals treated with levothyroxine for > 6 months, may solely represent overt hypothyroidism in real clinical practice. After adjustment for various comorbidities that affect mortality, hypothyroidism was still associated with 14% excess mortality when compared with controls. In line with our findings, Huang et al. also reported increased mortality in older (>65 years) hypothyroid subjects taking thyroid hormones when compared with non-hypothyroid individuals (12). Our results are also in agreement with those of recent meta-analyses (14, 19). A meta-analysis of 55 cohort studies, with no restrictions on age or degree of hypothyroidism, showed that hypothyroidism was associated with a higher risk of all-cause mortality than the euthyroid state (relative risk, 1.25; 95% CI: 1.13–1.39) (14). Another meta-analysis of 27 cohort studies focusing on the elderly population observed a significant association with increased all-cause mortality in patients with overt hypothyroidism, but not in those with subclinical hypothyroidism (19).

There are several plausible explanations for the increased risk of mortality in levothyroxine-treated hypothyroid patients in this study. First, it is possible that patients with severe hypothyroidism who were untreated for a long time have been included, although the medical records or thyroid function tests of enrolled patients in this study are not known. Second, treatment with levothyroxine may not fully reverse CVD risk factors that lead to increased mortality. Third, it is possible that hypothyroidism has either been over- or under-treated.

In the present study, patients with hypothyroidism had significantly higher prevalence of comorbidities than the control group. First, the higher prevalence of comorbidities in hypothyroid patients can plausibly be explained by the biological action of hypothyroidism itself. Hypothyroidism is known to induce many effects on the cardiovascular system, such as systolic and diastolic dysfunction, endothelial dysfunction, atherogenic lipid profiles, hypertension, and insulin resistance (6–10). A number of population studies have reported higher prevalence of atherosclerotic cardiovascular events and heart failure in hypothyroid patients (13–15, 28, 33). In addition, decreased hemodynamics in the circulatory system lead to declines in GFR in severe hypothyroidism (39). Second, it may be explained by the surveillance effect. Patients with comorbidities are more likely to visit clinics and undergo thyroid function tests than the subjects without comorbidities. Therefore, hypothyroidism is more likely to be detected in individuals with underlying disease.

Previous studies have suggested that the association between hypothyroidism and mortality is dependent on underlying comorbidities, especially CVD (13, 16). Studies in populations with high underlying CVD risk have shown that hypothyroidism is associated with higher all-cause and cardiovascular mortality (13, 23, 36, 37, 43). In a meta-analysis by Rodondi et al., the association between subclinical hypothyroidism and mortality did not differ according to pre-existing CVD (31). In the present study, we found that hypothyroidism is associated with higher all-cause mortality, with or without underlying CVD, although the association seemed to be more significant in the presence of CVD. Considering the impact of hypothyroidism on atherosclerosis, cardiac contractility, and arrhythmia, its association with higher mortality is plausible, particularly in populations with underlying CVD.

The risk of mortality in hypothyroidism may differ by age and sex (18, 20, 24, 28, 32, 34, 38). Previous studies have suggested that the association between hypothyroidism and mortality is less evident in individuals aged > 65 years (20, 32, 34, 44). Recently, Peng et al. found that the use of thyroid hormone replacement was not associated with all-cause mortality in patients aged > 65 years with subclinical hypothyroidism (38). The Leiden 85+ study, which enrolled individuals aged 85 years and older, indicated a decreased risk of cardiovascular and all-cause mortality with higher TSH levels (18), but these findings have not been replicated. Some studies have found increased risk of mortality only in male subjects with subclinical hypothyroidism (24, 28) while others have not (30). In the present study, the increased risk of mortality in hypothyroidism was consistently significant in age- and sex-stratified analyses, with the HR for mortality being higher in the younger age group and in men, which partly corroborated the results of previous studies.

In the present study, the mortality rate of hypothyroid patients increased regardless of age, sex, and CVD risk, but the HR for mortality decreased as the duration of thyroid hormone treatment increased. There are several plausible explanations for the higher mortality risk observed during initial treatment for hypothyroidism. First, the index date when hypothyroid patients were enrolled was defined as the date of first prescription of levothyroxine. Given that some hypothyroid patients may have been untreated for long periods of time before initiating thyroid hormone, there is a possibility that a large number of patients with hypothyroidism died during the early periods of therapy due to comorbidities associated with hypothyroidism. Second, mortality might increase during the early period of treatment due to under- or over-treatment. Thyroid function is more likely to be stable over a longer period of treatment; therefore, the mortality risk of hypothyroidism may be similar to that of the general population if euthyroid state is maintained. Thayakaran et al. found that hypothyroidism did not affect mortality when TSH concentrations were within the recommended normal limits (45). However, this finding was not confirmed in the present study owing to the absence of individual thyroid function tests.

The main strengths of our study include the large hypothyroid cohort of 501,882 individuals, the use of real-world data from primary care settings, and access to complete follow-up data. However, this study had several limitations. The diagnoses of hypothyroidism used in the study were based on administrative claims data, which may be less accurate than diagnoses based on biochemical data, as in other registry-based studies. It is possible that the hypothyroidism cohort includes patients with subclinical hypothyroidism who were asymptomatic but received a levothyroxine prescription after undergoing thyroid function tests. However, since the hypothyroidism cohort only targeted patients who took levothyroxine for more than 6 months, patients who took levothyroxine temporarily were likely to have been excluded. It is also possible that some patients with subclinical hypothyroidism were classified in the control cohort because the prevalence of subclinical hypothyroidism is about 3% in the general Korean population (46). Therefore, misclassification affects both the hypothyroidism cohort and the control cohort, and thus most likely does not affect our mortality risk estimates. It was impossible to obtain precise information concerning the timing of onset or duration of hypothyroidism, as was included in previous registry-based studies. Although thyroid function tests and the medical records of patients enrolled in the present study were unknown, patients with severe hypothyroidism who were untreated for long periods of time may have been included. Whether levothyroxine treated patients recovered to euthyroid state is unknown. However, we deduce that mortality tends to decrease in levothyroxine treated hypothyroid patients, as thyroid function is likely to be stable over time, because hazard ratios for mortality decreased as the duration of thyroid hormone treatment increased in our study. Lastly, although we adjusted for various underlying comorbidities, data for other potential confounders such as smoking status, body mass index, and alcohol consumption were not available.

In conclusion, in this nationwide, population-based cohort study we found that all-cause mortality was significantly higher in levothyroxine-treated hypothyroid patients than in non-hypothyroid controls. This association remained significant regardless of the age, sex, and cardiovascular disease risk. Further prospective cohort studies on the effects of hypothyroidism and levothyroxine treatment on mortality are warranted.

## Data Availability Statement

Data are available through the Health Insurance Review and Assessment Service in Korea 279 (HIRA). Researchers who wish to access the data can apply at (https://www.hira.or.kr).

## Ethics Statement

The studies involving human participants were reviewed and approved by Health Insurance Review and Assessment Service in Korea (Approval no. 2020-069). The ethics committee waived the requirement of written informed consent for participation.

## Author Contributions

SS and GS designed the study and wrote the manuscript. GS had access to all the data. SS and GS analyzed the data. JC was responsible for the decision to submit the manuscript. All authors contributed to the article and approved the submitted version.

## Funding

This work was supported by the Samjung Scholarship Foundation and by the faculty grant of Myongji Hospital (2002-09-07) to SS.

## Conflict of Interest

The authors declare that the research was conducted in the absence of any commercial or financial relationships that could be construed as a potential conflict of interest.
